# Brentuximab vedotin as monotherapy or combination therapy for cutaneous peripheral T cell lymphoma – a retrospective study

**DOI:** 10.1111/ddg.15826

**Published:** 2025-08-13

**Authors:** Caroline Glatzel, Inga Hansen‐Abeck, Nina Booken, Johannes Düll, Matthias Goebeler, Patrick Schummer, Marion Wobser

**Affiliations:** ^1^ Department of Dermatology Venereology and Allergology University Hospital Würzburg Würzburg Germany; ^2^ Department of Dermatology and Venereology University Medical Center Hamburg‐Eppendorf Hamburg Germany; ^3^ Department of Internal Medicine II Würzburg University Hospital University of Würzburg Würzburg Germany

**Keywords:** brentuximab vedotin, brentuximab vedotin mono‐ and combination therapy, cutaneous CD30‐positive peripheral T cell lymphomas, Cutaneous peripheral T cell lymphomas (PTCL)

## Abstract

**Background:**

Cutaneous peripheral T cell lymphomas (PTCL) are rare and show an aggressive course with limited response to therapy. The efficacy of brentuximab vedotin in cutaneous peripheral T cell lymphomas has not yet been systematically investigated.

**Patients and methods:**

In this retrospective analysis, we evaluated brentuximab vedotin as monotherapy or in combination therapy in patients with cutaneous CD30‐positive peripheral T cell lymphoma (n  =  9).

**Results:**

Overall, the therapy showed good efficacy and acceptable tolerability. One patient achieved an almost complete response, five had a partial response, and two had a mixed response as best overall response. The median time to response was 31.5 days (interquartile range 12–53). The median duration of response was short at 4.3 months. Median overall survival from initiation of brentuximab vedotin was 15.2 months; four of nine patients died from advanced lymphoma and one patient died from an unrelated cause.

**Conclusion:**

Brentuximab vedotin as monotherapy or combination therapy is a rapidly effective and tolerable treatment option for patients with cutaneous PTCL, although the duration of response is short.

## INTRODUCTION

Cutaneous peripheral T cell lymphomas (PTCL) represent a very rare subtype of primary cutaneous lymphomas and are often characterized by an aggressive clinical course with typically only short‐term responses to therapy.^1^ Polychemotherapy, usually following the CHOP regimen (cyclophosphamide, doxorubicin, vincristine, predniso[lo]ne), is commonly used.^2^, ^3^

Since 2017, a systemic therapy with brentuximab vedotin (BV)^4^, ^5^ has been approved for CD30‐positive cutaneous T cell lymphomas as second‐line therapy and shows high efficacy with acceptable tolerability in mycosis fungoides (MF), Sézary syndrome and CD30‐positive lymphoproliferations.^6^, ^7^

In the pivotal ALCANZA study (NCT01578499), systemic therapy with BV proved to be clearly superior to the control group (systemic therapy with methotrexate or bexarotene) in key endpoints. 54.7% of patients in the BV arm achieved an objective response (ORR4) lasting at least 4 months, compared to 12.5% in the control group.^8^ 17.2% of BV patients achieved a complete response (CR), compared to 1.6% in the control arm. The median progression‐free survival (PFS) in the BV arm was 16.7 (95% confidence interval [CI] 15.4–21.6) months compared to 3.5 (95% CI 2.4–4.6) months in the control group. At 14.2 months, the time to next tumor therapy (TTNT; time to next treatment) was significantly longer than the 5.6 months in the control group.^7^ Patients with cutaneous PTCL were not included in the ALCANZA study, although data on CD30 expression are also available for this lymphomatous entity.^9^

Although approved for cutaneous CD30‐positive PTCL, the efficacy of BV as monotherapy or combination therapy has not yet been systematically studied. We therefore present clinical data from nine patients with CD30‐positive cutaneous PTCL from two centers (Hamburg, Würzburg) who received monotherapy or combination therapy with BV.

## PATIENTS AND METHODS

In this retrospective analysis, the treatment regimen, tolerability and efficacy of treatment with BV as monotherapy or combination therapy in patients with CD30‐positive PTCL were investigated (n  =  9). The corresponding data on diagnosis, prior/follow‐up therapies, treatment regimen, response and adverse events were extracted from the electronic medical records of the two participating centers (Hamburg, Würzburg).

Response to therapy was defined as: *(1)* complete response (CR) with complete resolution of all tumor parameters of cutaneous PTCL; near complete response (nearCR) with complete resolution of all clinically known tumor parameters of cutaneous PTCL except singular/localized cutaneous lesions; *(2)* partial response (PR) as a decrease in all measurable tumor parameters by ≥ 30% with no evidence of progressive disease; *(3)* mixed response (MR) with simultaneous regression and progression of visible and measurable tumor parameters; *(4)* stable disease (SD) if neither CR/PR nor progression has been achieved in all lesions affected by the disease; and *(5)* progressive disease (PD) with an increase in size of the known lesion by ≥ 20% or tumor progression in an organ system (e.g. cutaneous or nodal/visceral manifestations).

The best overall response (BOR) was defined as the best response after initiation of BV. Progression‐free survival was defined as the period from initiation of BV therapy to disease progression and calculated using the Kaplan‐Meier method. Censoring was performed at death or last follow‐up. The median follow‐up time was calculated using the inverted Kaplan‐Meier approach (data cut‐off date June 30, 2024). All data analyses were performed using R (version 4.2.1; R packages: survival, survminer, ggplot2, ranger, prodlim).

## RESULTS

In this retrospective data analysis, nine patients with CD30‐positive cutaneous PTCL from two skin tumor centers (Würzburg, Hamburg) were included. Seven patients had PTCL without further specification (PTCL‐NOS) and two cutaneous follicular T‐helper cell lymphoma (FTHZL). The disease stage was advanced in the majority of cases with generalized skin involvement (stage T3 according to EORTC/ISCL).^10^ All patients had previously received at least one systemic therapy. In the participating skin tumor centers, there were no patients who were excluded from treatment with BV due to comorbidities. At the initiation of BV therapy, the patients had a median age of 53 years (range 31–77 years). The immunohistochemically recorded CD30 expression of the cutaneous manifestations was variable (≤ 10% in 6 patients, 30% in 2 patients and 60% in 1 patient) (Table 1).

**TABLE 1:** Baseline characteristics of PTCL patients.

Patient‐ID	Gender	Age at the start of BV	CD30 expression	Tumor stage	Interval between initial diagnosis and start BV [months]	System therapies before start of BV (in chronological order)
1	Female	77	< 5%	T3bN2M0	94	MTX, bexarotene
2	Male	67	10%	T3bN2M0	13	CHOP
3	Female	53	5%	T3bN0M0	92	Bexarotene, MTX, CHOP, bexarotene + oral PUVA, gemcitabine
4	Male	52	5%	T3bN1M0	19	Bexarotene
5	Male	50	60%	T3bN0M0	8	MTX
6	Male	51	≤ 5%	T3bN0M0	71	MTX, gemcitabine
7	Male	68	≤ 5%	T3bN0M0	216	CHOP, DHAP, MTX
8	Male	65	30%	T3bN2M0	5	Hydroxyurea
9	Female	31	30%	T3N3M0	40	Methotrexate, gemcitabine, CHOP

*Abbr*.: BV, brentuximab vedotin; MTX, methotrexate; CHOP, cyclophosphamide, doxorubicin, vincristine, predniso(lo)n; PUVA, psoralen + UVA; DHAP, dexamethasone, cytarabine, cisplatin.

Brentuximab vedotin was administered either as monotherapy (3/9), simultaneously with localized radiotherapy (4/9) or in combination with cyclophosphamide, doxorubicin and prednisolone (CHP) (2/9). Patients received a median of five cycles of BV (range 3–13), with a minimum of three doses administered. Among these treatment modalities, BV achieved good efficacy (nearCR in 1/9, PR in 5/9, MR in 2/9 patients, PD in 1/9 [primary treatment failure]). Patients with high CD30 expression levels (30% [n  =  2], > 60% [n  =  1]) did not show a better response to BV therapy than patients with significantly lower CD30 expression levels (Tables 1, 2).

**TABLE 2:** Therapy and response. Response to therapy was defined as: (a) complete response (CR) with complete resolution of all clinical tumor parameters; near complete response (nearCR) with resolution of all tumor parameters except for a small lesion; (b) partial response (PR) with regression of tumor parameters by ≥ 30% without progression; (c) mixed response (MR) with simultaneous regression and progression; (d) stable disease (SD) with no CR/PR or progression; (e) progressive disease (PD) with ≥ 20% increase in size or tumor progression in one organ system.

Patient‐ID	BV monotherapy/combination therapy	Best overall response	Regression of lesions compared to start BV	Follow‐up therapy (in chronological order)	Status at last follow‐up
1	BV+RTX (30 Gy)	Near CR	99%	Bexarotene	Died of lymphoma
2	BV	PR	≥30%	NA	Lost to follow‐up
3	BV	MR	≥30%	None	Died of lymphoma
4	BV	PD	None	NA	Lost to follow‐up
5	BV+RTX (6 Gy)	PR	≥30%	CHOP	Died of lymphoma
6	BV+CHP	PR	30%	none	Died of lymphoma
7	BV+CHP	MR	30%	Mogamulizumab+ TSEBT	Alive
8	BV+RTX (30 Gy)	PR	50%	None	Died of other causes
9	BV+RTX (22 Gy)	PR	75%	Allogeneic stem cell transplantation	Alive

*Abbr*.: BV, brentuximab vedotin; RTX, radiatio; CHP, cyclophosphamide, doxorubicin, prednisolone; NA, not specified; CHOP, cyclophosphamide, doxorubicin, vincristin, predniso(lo)n; TSEBT, total skin electron beam radiation.

**FIGURE 1:**
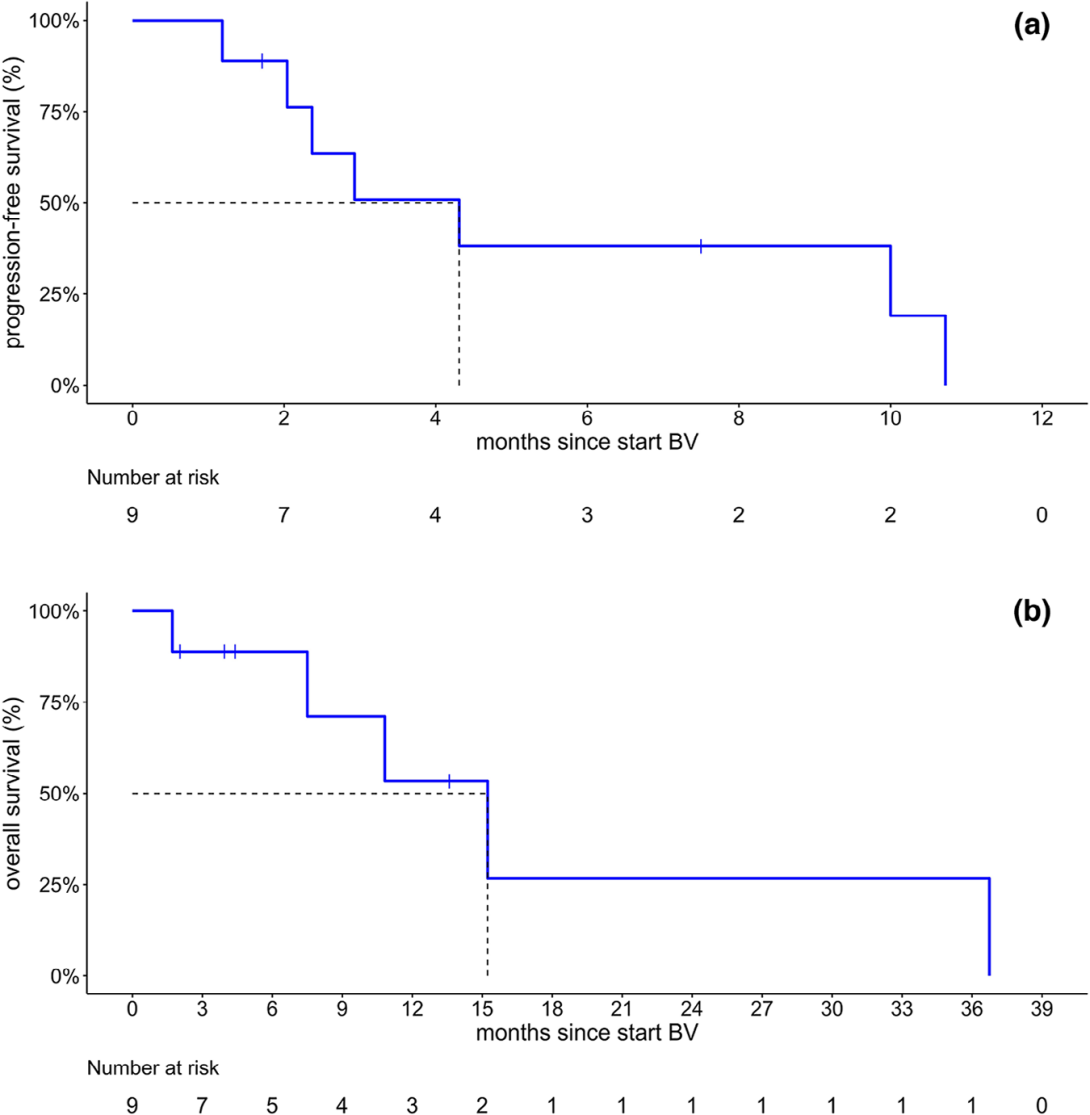
Therapeutic outcome after treatment with brentuximab vedotin (BV) as monotherapy or combination therapy. (a) Kaplan‐Meier plot of progression‐free survival for the overall cohort (n  =  9). Median progression‐free survival since initiation of brentuximab vedotin as monotherapy or combination therapy: 4.3 months (95% CI 2.4–NA). (b) Kaplan‐Meier plot of overall survival for the overall cohort (n  =  9). Median overall survival since initiation of BV: 15.2 months (95% CI 7.5–NA).

The median time to treatment response (CR/PR) was short, at approximately 1 month (31.5 days; interquartile range 12–53 days). After a median observation period of 13.6 months (range 4.4–NA), the median PFS after initiation of BV was 4.3 months (95% CI 2.4–NA) (Figure 1a). Median overall survival was 9.0 (95% CI 6.1–NA) years from initial diagnosis and 15.2 (95% CI 7.5–NA) months from initiation of BV (Figure 1b). The 1‐year overall survival since initiation of treatment with BV was 53% (25–100%), after 2 years it was 27% (6–100%). At the time of the last data collection, four out of nine patients had died due to progression of their lymphoma. Three of these deceased patients showed disease progression with nodal or visceral manifestation, one patient showed cutaneous progression. Another patient died secondary to a cardiac event.

Radiotherapy was administered concurrently with ongoing BV therapy in cases of newly developed or primary or secondary treatment‐resistant skin tumors. In one patient, early irradiation of nodal lymphoma manifestations was carried out in addition to BV administration. No patient received whole skin irradiation (TSEBT). All patients with cutaneous PTCL responded to combination therapy with localized radiotherapy (4/4). Three out of four patients achieved a PR and one patient a nearCR as best response. In four of nine cases, BV therapy was followed by subsequent treatment (Table 2); one patient who achieved a partial response under BV subsequently underwent allogeneic stem cell transplantation and had a survival time of 21 months from initiation of BV therapy.

During treatment with BV, seven out of nine patients experienced adverse events known for BV of any severity according to CTCAE (peripheral polyneuropathy (PNP): n  =  6/9, hematotoxicity: n  =  5/9) (Table 3). Severe adverse events (≥ grade 3 according to CTCAE) were observed in four of nine patients, including neutropenia, lymphopenia, and peripheral neuropathy (PNP). Treatment did not have to be discontinued prematurely in any patient due to side effects. One patient developed a tumor lysis syndrome under combined therapy of BV and simultaneous localized radiotherapy with a high cutaneous/nodal tumor burden. There were no deaths due to side effects. There was no evidence of unusual or unexpected toxicities or potentiation of side effects due to combination therapy.

**TABLE 3:** Adverse effects during therapy with brentuximab vedotin.

Adverse events	Patients n/n (%)
Any grade	7/9 (78)
> 1 side effect	5/9 (56)
≥ Grade 3	4/9 (44)
**Involvement of organ systems**	
Neurologic (polyneuropathy)	6/9 (67)
Hematologic	5/9 (56)
Liver/Pancreatic	2/9 (22)
Renal	1/9 (11)
Other*	3/9 (33)

Due to rounding, percentages may not add up to 100. *Other documented adverse reactions include: soor esophagitis, radiation dermatitis, fatigue, weight loss, SARS‐CoV‐2 infection, ulceration of tumor nodes.

## DISCUSSION

The treatment of cutaneous PTCL poses a therapeutic challenge due to its aggressive course with a high risk of systemic dissemination and correspondingly high mortality.^11^, ^12^ Preferred (poly)chemotherapies in PTCL usually show a short response with recurrences within a few months.^2^, ^11^, ^13^ The treatment of a patient with cutaneous PTCL often remains a case‐by‐case decision.^3^ Randomized studies on the use of BV in cutaneous PTCL have not yet been conducted. In this retrospective survey, we therefore evaluated BV as monotherapy or combination therapy in patients with CD30‐positive PTCL (n  =  9) with regard to treatment regimen, tolerability and efficacy.

In our cohort, BV showed rapid effectiveness and good tolerability as monotherapy or combination therapy.

BV treatment, initiated as second‐line therapy and in five cases as third‐line or later‐line therapy, led to a response after a mean of 31.5 days in the patient population presented here. This observation is particularly valuable in view of the rapid progression of the disease, as prompt diagnosis, early initiation of treatment and a fast response can improve the prognosis.^3^ BV did not exhibit an unusual side effect profile in patients with PTCL, either as monotherapy or in combination therapy.^7^ Side effects were consistent with those seen in other studies.^7^, ^11^ Peripheral neuropathy occurred in 67% of patients in our analysis, compared to 69% in the ALCANZA trial. High‐grade adverse events (≥ grade 3 according to CTCAE) were observed in four of nine patients (44%) in our cohort, compared to 41% in the ALCANZA study.^7^

The good tolerability of BV in both monotherapy and combination therapy settings, the overall manageable side effect profile, and the absence of treatment discontinuations due to adverse events in our cohort support the consideration of BV as a potential treatment option for PTCL in the future.

The initial response to BV was favorable in the majority of cases. Six of nine patients achieved a complete or partial response, which is comparable to the objective response rate (ORR; CR and PR) reported in the ALCANZA trial (BV arm: 65.6%; control arm: 20.3%).^8^ In the ALCANZA study, patients with CD30 expression ≤ 10% did not show a poorer response to BV than those with higher CD30 expression.^14^ In our analysis as well, higher CD30 expression did not necessarily correlate with a better or more durable response.

Despite the rapid and favorable response to BV and its manageable side effect profile, patients with PTCL continued to have a poor prognosis in our analysis. With a median PFS of 4.3 months, the duration of response in PTCL was limited (Figure 1a). In contrast, patients with MF and pcALCL in the ALCANZA study achieved a nearly fourfold longer median PFS of 16.7 months.^7^

The majority of patients in our analysis were in advanced stages of disease with generalized skin involvement (stage T3 according to EORTC/ISCL). In the literature, these advanced stages of cutaneous PTCL were also associated with a significantly worse prognosis than localized manifestations (p < 0.034).^2^, ^3^, ^11^

Like BV, the currently available treatment options for PTCL patients show limited long‐term efficacy.^1^ Half of the patients survived for less than 15 months after initiation of BV therapy. However, due to the rapid and good response, the use of BV as a remission‐inducing therapy prior to stem cell transplantation would be conceivable.^15^ This is further illustrated by a patient in our cohort who achieved a partial response with BV and subsequently underwent allogeneic stem cell transplantation. His survival time from initiation of therapy was 21 months.

The efficacy of BV in cutaneous PTCL with respect to progression‐free and overall survival should be evaluated in prospective randomized clinical trials, including its use in optimized combination regimens and as a remission‐inducing therapy prior to stem cell transplantation. In our view, it is therefore essential to collect clinical cases of cutaneous PTCL in registries in order to improve patient care in the long term, despite the currently limited treatment options. Moreover, there remains a substantial need for novel therapeutic approaches in cutaneous PTCL.

## CONFLICT OF INTEREST STATEMENT

C.G. received honoraria from Recordati Rare Diseases outside the submitted work. I.H. received travel grants from Kyowa Kirin outside the submitted work. M.G. received honoraria, all outside the subject matter of this publication, for work on advisory boards or for lectures from Almirall, Argenx, Biotest, GSK, Janssen, Leo Pharma, Lilly, Novartis, and UCB. M.W. received honoraria and travel grants outside the submitted work from TAKEDA, Recordati Rare Diseases, Stemline Therapeutics, and Kyowa Kirin. All other authors declare no conflict of interest.
